# Maternal folic acid supplement timing and learning difficulties in children assessed at ages 4 to 7 years

**DOI:** 10.3389/fpubh.2026.1937716

**Published:** 2026-09-25

**Authors:** Hanbin Wu, Zhuoyuan Xu, Yingren Luo, Chengchen Zhang

**Affiliations:** 1 Department of Obstetrics and Gynaecology, The Chinese University of Hong Kong, Hong Kong, Hong Kong SAR, China; 2 National Institute for Family Planning, Beijing, China; 3 Department of Cardiology, National Children’s Medical Center, Shanghai Children’s Medical Center, Shanghai Jiao Tong University School of Medicine, Shanghai, China; 4 Center of Clinical Research, National Children’s Medical Center, Shanghai Children’s Medical Center, Shanghai Jiao Tong University School of Medicine, Shanghai, China

**Keywords:** cohort study, folic acid, learning difficulties, prenatal nutritional supplementation, time-to-treatment initiation

## Abstract

**Background:**

Periconceptional folic acid supplementation prevents neural tube defects and may influence offspring neurodevelopment, but evidence for domain-specific learning difficulties remains unclear. We examined whether maternal-reported first-trimester initiation of folic acid supplementation was associated with parent-reported learning difficulties in offspring assessed at ages 4–7 years.

**Materials and methods:**

This retrospective cohort study used data from the Dutch Perined-Lifelines birth cohort. We excluded preterm birth (<37 weeks), high-dose folic acid exposure (4.0 mg), and supplementation initiated after the first trimester. Exposure was retrospective maternal-reported folic acid supplementation timing and categorized as before conception/prepregnancy through the first trimester and first trimester only. Outcomes were parent-reported binary indicators of poor concentration, reading disability, difficulty with calculating, and difficulty keeping up at school without clinical diagnoses or standardized neuropsychological assessments. Logistic regression estimated odds ratios (ORs) and 95% confidence intervals (CIs), adjusting for child age, sex, body rate, stress rate, and preschool sleeping problems.

**Results:**

Of 3,216 children, 2,314 (71.9%) were in the preconception group and 902 (28.1%) in the first-trimester-only group. In adjusted models, first-trimester-only supplementation was not associated with poor concentration, reading disability, or difficulty keeping up at school but was nominally associated with higher odds of parent-reported difficulty with calculating (OR = 1.60; 95% CI: 1.02–2.50).

**Conclusion:**

Maternal-reported first-trimester initiation of folic acid supplementation was nominally associated with parent-reported difficulty with calculating assessed at ages 4–7, but the retrospective observational design, potential misclassification, and residual confounding preclude causal interpretation. Prospective studies with biomarkers and standardized developmental assessments are warranted.

## Introduction

Folic acid supplementation during the periconceptional period is a well-established public health strategy for preventing neural tube defects (1). International recommendations emphasize that women who are planning pregnancy or may become pregnant should take folic acid before conception and continue during early pregnancy because neural tube formation and closure occur very early in gestation (2). Beyond the prevention of major structural congenital anomalies, growing attention has been paid to whether maternal folate status may also influence long-term offspring neurodevelopment.

Folate is involved in one-carbon metabolism, nucleotide synthesis, methylation reactions, and epigenetic regulation, all of which are important for embryonic growth and early brain development (3, 4). Because the preconception period and first trimester represent sensitive windows for fetal neurodevelopment, the timing of folic acid supplementation may be biologically relevant (5). Previous studies have reported associations between maternal folic acid supplementation and several offspring neurodevelopmental outcomes, including language development and autism spectrum disorder (6–8). However, evidence across broader cognitive and behavioral outcomes remains inconsistent (9).

Childhood learning difficulties are heterogeneous and may involve distinct neurocognitive processes. Poor concentration, reading disability, calculating disability, and difficulty keeping up at school may reflect partly different developmental pathways. In particular, mathematical and calculating abilities rely on working memory, executive control, visuospatial processing, and fronto-parietal neural networks, which may be especially sensitive to early neurodevelopmental influences. Therefore, examining different learning difficulty phenotypes separately may help clarify whether the association between maternal folic acid supplementation and offspring learning outcomes is domain-specific rather than general.

Using data from the Perined-Lifelines linked birth cohort (10), we aimed to explore whether the timing of maternal folic acid supplementation, as self-reported, was associated with parent-reported learning difficulties among children aged 4–7 years.

## Methods

### Study design and data source

This study was conducted as a retrospective cohort analysis using data from the Dutch Lifelines Cohort Study. Lifelines is a large population-based, multigenerational cohort study and biobank in the northern Netherlands that investigates the complex interplay of environmental, phenotypic, and genomic factors in the development of chronic diseases and healthy aging (10). Between 2006 and 2013, adults were recruited through general practitioners, after which participating individuals could invite family members, including parents, partners, and children. Family connections among participating individuals were identified using information from the Dutch citizen registration and self-reported information, with genotyping information used where available. Mother–child pairs in the present study were identified using the available family-linkage information within Lifelines (11). Participants attended Lifelines research sites for physical examinations, including pulmonary function testing, electrocardiography, and cognitive assessments, and completed detailed questionnaires. At baseline, data were collected from 167,729 participants aged from 6 months to 93 years. The cohort is planned for long-term follow-up over at least 30 years, with questionnaires administered approximately every 1.5 years and physical examinations conducted every 5 years. Data collection covered physical and mental health, lifestyle, environmental exposures, and related domains.

### Study population

Of the 16,555 children who participated in the Lifelines baseline assessment, mother–child pairs were identified based on maternal self-reported folic acid supplementation during pregnancy and available offspring data. Those children were excluded if they: (1) were born before 37 weeks of gestation; (2) had mothers who used high-dose folic acid supplementation (4.0 mg) during pregnancy; or (3) had mothers who initiated folic acid supplementation only after the first trimester. Following these exclusions, a total of 3,216 children were included in the baseline analysis. The participant selection procedure is presented in Figure 1.

**Figure 1:**
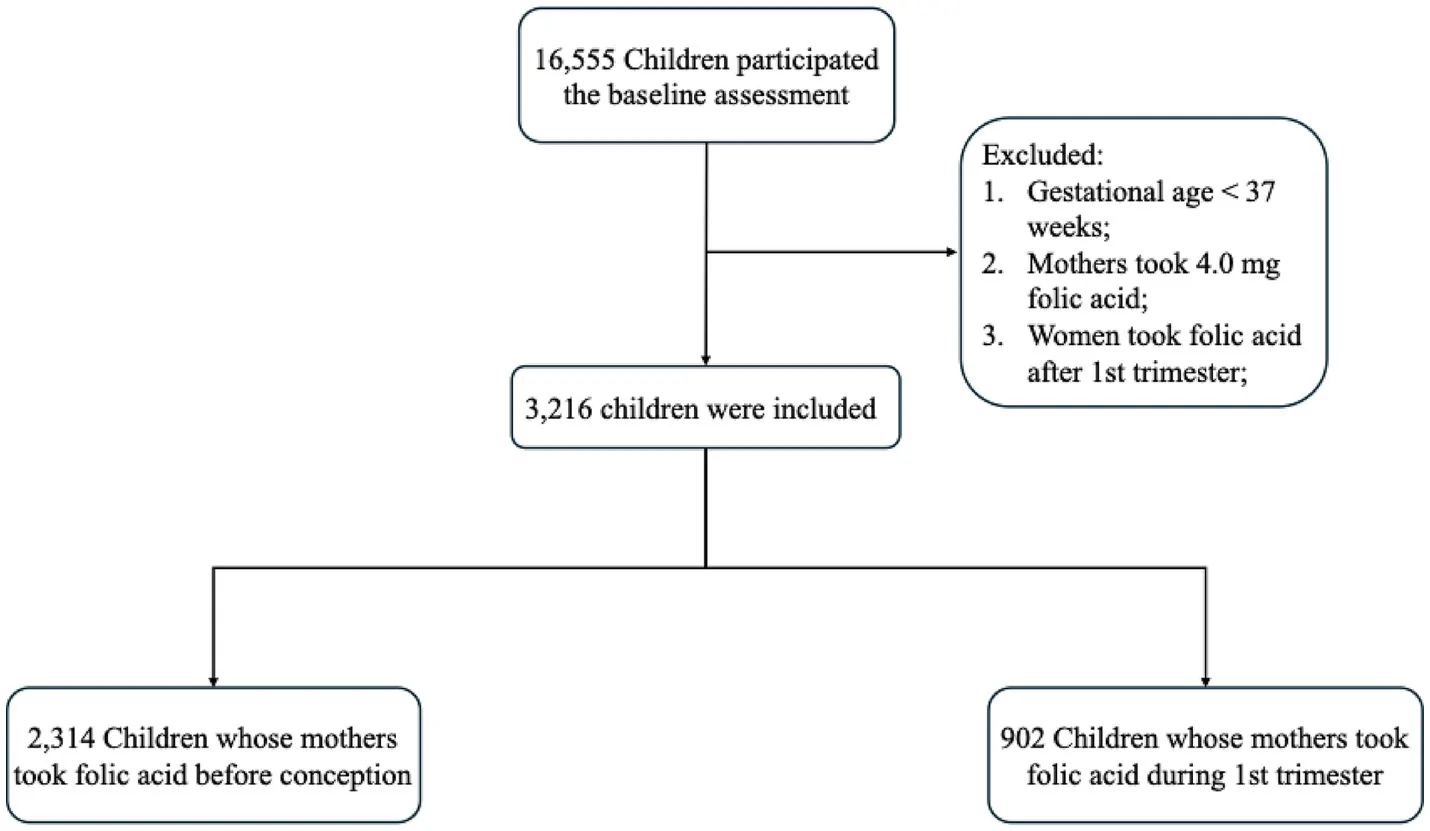
Flowchart of the participants’ selection.

### Exposure and outcome assessment

The exposure was maternal self-reported timing of folic acid supplementation, categorized as use before conception or from pre-pregnancy through the first trimester versus (vs.) use during the first trimester only.

Outcomes were parent-reported indicators of poor concentration, reading difficulty, difficulty with calculating, and difficulty keeping up at school, each treated as a binary variable. These outcomes should not be interpreted as standardized clinical diagnoses, including developmental dyscalculia.

### Covariates

The adjusted models included the following covariates measured at baseline: child age, sex, body rate, stress rate, and preschool sleeping problems. Child age and stress rate were treated as continuous variables. Sex was categorized as male or female. Body rate was categorized as normal, thin, or overweight. Preschool sleeping problems were defined based on parental report at 4–5 years of age and were treated as a binary variable.

### Statistical analysis

Baseline characteristics were compared using *t*-tests or Kruskal–Wallis tests for continuous variables and the chi-squared tests or Fisher’s exact tests for categorical variables. Logistic regression estimated ORs and CIs. Adjusted models included child age, sex, body rate, stress rate, and preschool sleeping problems. Analyses were conducted using complete cases for each outcome. To address the multiple comparison issues across the four outcomes, Benjamini–Hochberg false discovery rate (FDR)-adjusted q values were calculated. Data analyses were performed in R version 4.2.2 (R Foundation for Statistical Computing). Two-sided nominal *p*-values of < 0.05 were reported, and FDR-adjusted q values of <0.05 were considered statistically significant after correction for multiple testing.

## Results

### Baseline characteristics

Of the 3,216 children included in the analysis, 2,314 children (71.95%) were born to mothers who used folic acid before conception or from prepregnancy to the first trimester, and 902 children (28.05%) were born to mothers who used folic acid during the first trimester only. The comparison of baseline characteristics by timing of maternal folic acid supplementation is shown in Table 1. Compared with children in the preconception/preconception-plus-first-trimester group, those in the first-trimester-only group were slightly older at baseline and more likely to have been conceived naturally. Their mothers had higher rates of alcohol consumption and smoking across all three trimesters but were less likely to have undergone assisted reproduction, including artificial insemination and IVF/ICSI. Additionally, normal vaginal birth was more prevalent in the first-trimester-only group, whereas rates of assisted vaginal delivery and caesarean section were lower. No significant between-group differences were observed in gestational age, birth defects, breastfeeding, sex distribution, body rate, preschool sleeping problems, stress rate, or maternal educational level. With respect to learning outcomes at 4–7 years, poor concentration and reading disability were comparable between groups; however, parent-reported difficulty with calculating was more prevalent in the first-trimester-only group than in the preconception/prepregnnacy-initiation group (8.55% vs. 5.59%; *p* = 0.04).

**Table 1:** Baseline characteristics by timing of maternal folic acid supplementation.

Characteristics	Prior to pregnancy or from prepregnancy to first trimester	Only first trimester	*p*-value
Sample size	2,314	902	
Conception type			<0.001
Natural	2,183 (94.42%)	894 (99.22%)	
Artificial insemination	52 (2.25%)	2 (0.22%)	
IVF/ICSI	67 (2.90%)	3 (0.33%)	
Others	10 (0.43%)	2 (0.22%)	
Drinking in first trimester	116 (5.06%)	116 (13.05%)	<0.001
Drinking in second trimester	58 (2.52%)	46 (5.13%)	<0.001
Drinking in third trimester	76 (3.30%)	49 (5.48%)	0.006
Smoking in first trimester	370 (16.66%)	210 (24.42%)	<0.001
Smoking in second trimester	334 (15.11%)	188 (21.96%)	<0.001
Smoking in third trimester	316 (14.30%)	182 (21.24%)	<0.001
Gestational age (weeks)	39.87 ± 1.41	39.91 ± 1.52	0.424
Delivery type			<0.001
Normal vaginal birth	1,597 (69.04%)	684 (75.83%)	
Vaginal birth with use of forceps or vacuum pump	326 (14.09%)	107 (11.86%)	
Cesarean section	390 (16.86%)	111 (12.31%)	
Birth defects	132 (5.73%)	57 (6.40%)	0.528
Breastfeeding	1871 (81.17%)	737 (82.07%)	0.591
Children’s age at baseline (years)	7.25 ± 4.40	7.96 ± 4.46	<0.001
Boys	1,181 (51.04%)	436 (48.34%)	0.181
Poor concentration (4–7 years of age)*			0.416
Yes	238 (22.47%)	116 (24.52%)	
No	821 (77.53%)	357 (75.48%)	
Reading difficulty (4–7 years of age)*			0.697
Yes	83 (8.07%)	40 (8.83%)	
No	946 (91.93%)	413 (91.17%)	
Calculating difficulty (4–7 years of age)*			0.040
Yes	59 (5.59%)	40 (8.55%)	
No	997 (94.41%)	428 (91.45%)	
Difficult keeping up at school (4–7 years of age)*			
Yes	98 (9.18%)	52 (11.04%)	
No	969 (90.82%)	419 (88.96%)	
Body rate			0.762
Exactly right	1,305 (77.22%)	542 (77.54%)	
Thin	219 (12.96%)	84 (12.02%)	
Fat	166 (9.82%)	73 (10.44%)	
Problems falling asleep as a preschooler (4–5 year of age)	150 (13.99%)	63 (13.12%)	0.704
Stress rate	3.45 ± 2.22	3.64 ± 2.10	0.122
Unable to concentrate			0.178
Not at all	628 (61.51%)	259 (57.17%)	
A bit or sometimes	306 (29.97%)	144 (31.79%)	
Clearly or often	87 (8.52%)	50 (11.04%)	
Educational level			0.864
Preparatory secondary vocational education	23 (6.89%)	12 (6.98%)	
Primary education	3 (0.90%)	1 (0.58%)	
Preparatory secondary vocational education-theoretical pathway	65 (19.46%)	30 (17.44%)	
Preparatory secondary vocational education-other	31 (9.28%)	23 (13.37%)	
Senior general secondary education	84 (25.15%)	50 (29.07%)	
Pre-university secondary education with Latin and Greek	98 (29.34%)	45 (26.16%)	
Secondary vocational education	14 (4.19%)	8 (4.65%)	
Special-needs secondary education	4 (1.20%)	1 (0.58%)	
Practical training	1 (0.30%)	0 (0.00%)	
Higher vocational education	3 (0.90%)	0 (0.00%)	
Other type of education	8 (2.40%)	2 (1.16%)	

Values are numbers (percentages) unless otherwise indicated.

Stress rate indicates the parent-reported perceived stressfulness of the child’s life since the previous Lifelines questionnaire, rated from 1 to 10, with higher scores indicating greater stress.

Body rate was categorized using age- and sex-specific BMI categories as exactly right, thin, or fat.

Preschool sleeping problem was parent-reported and defined as any sleep-related problem at age 4 to 5 years, including difficulty falling asleep, night waking, nightmares, bedwetting, snoring, sleepwalking, or other sleep problems.

*Parent-reported learning outcomes represent questionnaire-based concerns and do not indicate clinical diagnoses.

### Association between timing of maternal folic acid supplementation and offspring learning difficulties

The associations between maternal-reported first-trimester initiation of folic acid supplementation and parent-reported learning-related outcomes at 4–7 years are presented in Table 2. Compared with the children whose mother used folic acid supplements prior to pregnancy or from prepregnancy to first trimester, first-trimester-only supplementation was not associated with poor concentration, reading disability, or difficulty keeping up at school. In contrast, maternal-reported first-trimester-only initiation was nominally associated with higher odds of parent-reported difficulty with calculation after covariate adjustment (adjusted OR: 1.60, 95% CI: 1.02–2.50, *p* = 0.039). However, after Benjamini–Hochberg correction across the four outcomes, this association did not remain statistically significant at an FDR of 5% and should therefore be interpreted as exploratory.

**Table 2:** Associations of maternal-reported folic acid supplementation timing with parent-reported learning difficulties in children assessed at ages 4–7 years.

Outcomes	Crude model		Adjusted model	
	OR (95% CI)	*p*-value	OR (95% CI)	*p*-value
Poor concentration*				
Folic acid before conception	1.00 Reference	…	1.00 Reference	…
Folic acid during first trimester only	1.12 (0.87, 1.44)	0.379	1.18 (0.89, 1.54)	0.241
Reading difficulty*				
Folic acid before conception	1.00 Reference	…	1.00 Reference	…
Folic acid during first trimester only	1.10 (0.74, 1.63)	0.623	1.11 (0.73, 1.67)	0.616
Calculating difficulty*				
Folic acid before conception	1.00 Reference	…	1.00 Reference	…
Folic acid during first trimester only	1.58 (1.03, 2.39)	0.032	1.60 (1.02, 2.50)	0.039
Keeping up problem at school*				
Folic acid before conception	1.00 Reference	…	1.00 Reference	…
Folic acid during first trimester only	1.23 (0.86, 1.74)	0.259	1.18 (0.80, 1.72)	0.385

Model was adjusted for children’s age, sex, body rate, stress rate, and preschool sleeping problems.

*Parent-reported learning outcomes represent questionnaire-based concerns and do not indicate clinical diagnoses.

Benjamini–Hochberg correction was applied across the four adjusted outcomes. The FDR-adjusted *q* value for parent-reported difficulty with calculating was 0.156.

## Discussion

In this retrospective cohort study, maternal-reported initiation of folic acid supplementation during the first trimester only, compared with supplementation beginning before conception or continuing from pre-pregnancy through the first trimester, was nominally associated with higher odds of parent-reported difficulty with calculating around age 7. However, no evident associations were observed for poor concentration, reading disability, or difficulty keeping up at school. Notably, this is an exploratory study, and these findings should not be interpreted as evidence that later supplementation initiation causes impaired mathematical ability or as evidence of a clinical diagnosis of dyscalculia.

These findings extend previous research on periconceptional folic acid beyond its established role in preventing neural tube defects (12, 13). Previous studies have reported associations between maternal folic acid supplementation and childhood neurodevelopmental outcomes, including language delay, autism spectrum disorder, psychomotor development, and cognitive performance (6, 7, 9, 14, 15). However, most previous studies did not evaluate domain-specific learning difficulties, and evidence specifically linking folic acid timing with mathematical development remains limited. Although folate has established roles in one-carbon metabolism, nucleotide synthesis, and methylation processes relevant to early brain development (16), and mathematical learning involves numerical processing, working memory, visuospatial function, and executive function (17), these biological considerations do not establish a mechanism for the observed association. Importantly, this study assessed maternal-reported supplement initiation timing rather than dietary folate intake, adherence, or biological folate status. Furthermore, the reported first-trimester-initiation group differed from the preconception-initiation group in maternal smoking, alcohol consumption, and reproductive characteristics, whereas the original adjusted models included selected child-level covariates. Thus, residual maternal and family-level confounding and outcome misclassification may explain the nominal association with parent-reported difficulty with calculating. This finding should therefore be considered exploratory and hypothesis-generating rather than evidence of a specific effect of folic acid timing on offspring mathematical ability.

Several limitations should be acknowledged. First, the retrospective observational design precludes causal inference. The timing of maternal folic acid supplementation and children’s learning difficulties were self- or parent-reported, and data on serum or red blood cell folate concentrations, standardized neurodevelopmental assessments, or clinical diagnoses were unavailable. Thus, exposure and outcome misclassification, as well as recall and reporting bias, cannot be excluded. Second, the study lacked detailed information on supplement adherence, dietary folate intake, home learning environment, maternal cognitive ability, and standardized neuropsychological assessments. Third, outcome-specific analyses were based on available cases, and selection bias related to missing outcome data could not be ruled out. Fourth, potential clustering of children within mothers was not accounted for. Finally, as multiple learning outcomes were examined, the observed association with parent-reported difficulty with calculating should be interpreted as exploratory. Prospective studies using detailed supplement use information, folate biomarkers, standardized developmental assessments, and comprehensive control of maternal and family-level factors are needed.

In summary, maternal-reported initiation of folic acid supplementation in the first trimester was nominally associated with parent-reported difficulty with calculating in this retrospective cohort. This exploratory finding should not be interpreted as evidence that later initiation of supplementation causes learning disability or reflects a definitive biological effect of folic acid exposure. Rather, it is consistent with existing recommendations emphasizing folic acid supplementation before conception and generates a hypothesis that requires confirmation. Replication in prospective cohorts with objective folate biomarkers, standardized developmental assessments, and careful control of maternal and family-level confounders is needed.

## Data Availability

The datasets presented in this article are not readily available because the data used in this study were obtained from the Lifelines Cohort Study (https://www.lifelines-biobank.com/). These data are not owned by the authors and are available from Lifelines for researchers who meet the criteria for access to confidential data. Information regarding data access procedures is available through the Lifelines Cohort Study data access process. Requests to access the datasets should be directed to https://www.lifelines-biobank.com/.
